# Voltage‐Gated Calcium Channel Blockers: Preclinical Insights, Clinical Applications, and Emerging Therapeutic Advances

**DOI:** 10.1002/alz70855_105395

**Published:** 2025-12-24

**Authors:** Nikhil Kalra

**Affiliations:** ^1^ MM college of Pharmacy, Ambala, Haryana, India

## Abstract

**Background:**

Neuronal excitability, together with neurotransmitter release and synaptic plasticity, rests upon active voltage‐gated calcium channels (VGCCs). Scientific evidence shows VGCC activity dysfunction results in epilepsy and neuropathic pain and neurodegenerative disease development. VGCC blockers have established themselves as promising treatments for these medical conditions, which requires us to investigate their complete preclinical along with clinical performance.

**Method:**

This research combines results from preclinical tests and clinical trials to understand VGCC blockers in terms of pharmacology, operation, and treatment effectiveness. Research scientists evaluated VGCC blocker properties by analysing findings from animal experiments and human clinical trials to determine their brain protection abilities and their utility in pain treatment and novel applications in psychiatric treatments and neurodegenerative diseases.

**Result:**

Laboratory investigations demonstrate that VGCC blockers control synaptic transmission while simultaneously decreasing brain cell toxicity and reducing inflammatory responses in the nervous system. As clinical research reveals, VGCC blockers can effectively manage hypertension as well as epilepsy and chronic pain, but scientists are finding new applications for these agents in treating Alzheimer's and Parkinson's disease. The development of new therapeutic treatments focuses on Cav2.2 and Cav3.2 VGCC subtype blockers to advance individualised medicine approaches.

**Conclusion:**

Multiple brain and heart diseases benefit from the healing potential of VGCC‐blocking agents. The combination of more targeted channel modulation approaches alongside advanced drug delivery techniques shows potential to enhance both the effectiveness and safety of treatments. Scientists need to conduct additional studies both to optimise therapeutic potential and investigate new treatment methods.

